# Predictors of mental well-being among family caregivers of adults with intellectual and developmental disabilities during COVID-19

**DOI:** 10.1192/bjo.2024.761

**Published:** 2024-10-28

**Authors:** Olivia Mendoza, Laura St. John, Gabriel Tarzi, Anupam Thakur, Johanna K. Lake, Yona Lunsky

**Affiliations:** Azrieli Adult Neurodevelopmental Centre, Centre for Addiction and Mental Health, Toronto, Canada; Azrieli Adult Neurodevelopmental Centre, Centre for Addiction and Mental Health, Toronto, Canada; and Department of Psychiatry, University of Toronto, Toronto, Canada

**Keywords:** Intellectual disability, developmental disability, pandemic, caregiving, mental health

## Abstract

**Background:**

Internationally, stresses related to the COVID-19 pandemic negatively affected the mental health of family caregivers of adults with intellectual and developmental disabilities (IDDs).

**Aims:**

This cross-sectional study investigated demographic, situational and psychological variables associated with mental wellbeing among family caregivers of adults with IDDs during the COVID-19 pandemic.

**Method:**

Baseline data from 202 family caregivers participating in virtual courses to support caregiver mental well-being were collected from October 2020 to June 2022 via online survey. Mental well-being was assessed using total scores from the Warwick-Edinburgh Mental Wellbeing Scale. Demographic, situational and psychological contributors to mental well-being were identified using hierarchical regression analysis.

**Results:**

Variables associated with lower levels of mental well-being were gender (women); age (<60 years old); lack of vaccine availability; loss of programming for their family member; social isolation; and low confidence in their ability to prepare for healthcare, support their family member's mental health, manage burnout and navigate healthcare and social systems. Connection with other families, confidence in managing burnout and building resilience and confidence in working effectively across health and social systems were significant predictors of mental well-being in the final regression model, which predicted 55.6% of variance in mental well-being (*P* < 0.001).

**Conclusions:**

Family caregivers need ways to foster social connections with other families, and support to properly utilise healthcare and social services during public health emergencies. Helping them attend to their needs as caregivers can promote their mental health and ultimately improve outcomes for their family members with disabilities.

The COVID-19 pandemic led to a significant strain on the global population and adults with intellectual and developmental disabilities (IDDs), such as autism or Down syndrome, have been negatively affected in particular. Many adults with IDDs require tailored approaches and accommodations for medical management, and often access a broad variety of social and healthcare services in their communities.^1^ COVID-19 restrictions resulted in a loss of programmes, resources and services for this under-resourced population,^2,3^ subsequently causing disruptions in integral routines, increased isolation and loss of invaluable support.^4,5^ Before the COVID-19 pandemic, adults with IDDs were more likely to have comorbid mental and physical health issues, receive less preventative healthcare and have higher rates of ambulatory care use.^6,7^ The pandemic and its associated disruptions exacerbated these pre-existing physical health disparities; adults with IDDs in various parts of the world experienced greater odds of being infected with COVID-19 and worse health outcomes following infection.^8–10^ The pandemic also had adverse impacts on the mental health of adults with IDDs; research has shown that adults with IDDs have poorer mental health compared to before the pandemic, and are seeking medical treatment more frequently for psychiatric issues.^4,11,12^

## The impact of COVID-19 on caregivers of adults with IDDs

Pandemic-related stressors also extended to family caregivers of adults with IDDs (hereinafter referred to as caregivers). Before the pandemic, caregivers faced an increased risk for poor physical and mental health outcomes,^13–15^ and these negative outcomes were more pronounced during COVID-19.^2,3^ Caregivers also faced an increased burden of care because of the significant loss of services and support that many adults with IDDs traditionally relied on (i.e. day services, respite care, social activities and educational/employment services).^2,3,16^ Many reported feeling unable to support the needs of their relatives, and observed increased behavioural concerns.^16–20^ Caregivers have also faced pandemic-related stressors that are unrelated to caregiving, such as loss of employment, financial hardship and reduced social interactions.^17,21^ All of these pandemic-related factors may have compounded and contributed to caregivers of adults with IDDs having higher levels of anxiety and depression than caregivers of neurotypical individuals.^2,3^

## Existing research on the impact of COVID-19 on caregivers of adults with IDDs

While research has investigated the impact of COVID-19 on the mental well-being of family caregivers of people with IDDs, there has been less attention on the demographic, psychological and situational variables associated with their mental well-being during this time. Furthermore, most of the studies published have focused on the family experience early in the pandemic. It is important to consider how ongoing pressures, the availability of vaccines, access to healthcare and structured activities and connections with other families have affected caregivers. An improved understanding of potential contributors to mental well-being may have implications in terms of which families should be prioritised for additional mental health support.

## Study objective

The purpose of this study was to identify demographic, situational and psychological predictors of mental well-being in a Canadian cohort of family caregivers of adults with IDDs during the COVID-19 pandemic. We leveraged data collected as part of two national intervention projects of family caregivers, conducted between fall 2020 and summer 2022.

## Method

### Design

The current study is a secondary analysis of data previously collected as part of two caregiver projects evaluating virtual intervention programmes to help them cope with pandemic challenges.^22,23^ To recruit study participants for the original studies, researchers contacted project partners and collaborating organisations representing four national and 12 provincial groups who shared flyers via email and social media, in addition to sharing information through the research centre's newsletter over a 3-week period before each cycle's start. Interested people could complete an online expression of interest form or send an email to speak with research staff.

Eligibility for participant inclusion was as follows: (a) a family member of an adult with an IDD who is 18 years of age or older, (b) lived in Canada, (c) provided informed consent and (d) had access to a telephone or internet.^22^ The course ran in five 6-week cycles from October 2020 to June 2022.

### Measures

Before starting the course, participants responded to an online survey that included items pertaining to demographic information, COVID-19 experiences, mental well-being and self-efficacy. For the purpose of this study, only the baseline data collected before the implementation of the course intervention were analysed.

Respondents indicated their experience with COVID-19-related changes using a three-point Likert scale (never, sometimes, a lot). Mental well-being was evaluated using the 14 item Warwick-Edinburgh Mental Wellbeing Scale (WEMWBS) questionnaire. This measure assesses feeling and functioning aspects of mental well-being using a five-point Likert scale, with available responses as follows: none of the time, rarely, some of the time, often, all the time. Scores range from 14 to 70 with a higher score indicating a higher degree of mental well-being. The scale's test–retest reliability was found to be 0.83 (*P* < 0.01) in the initial validation study.^24^ Confidence in four competencies related to healthcare and IDDs was measured through ‘self-efficacy’ variables. These were assessed using a numeric 100 point sliding scale adapted from previously published self-efficacy scales by Sockalingam et al,^25^ wherein a score of zero indicated ‘not confident’ and a score of one hundred indicated ‘very confident’.^22^

Survey responses were collected via REDcap (Vanderbilt University, TN, USA; https://www.project-redcap.org/). All participants provided written informed consent to have their responses analysed for research purposes. This study was approved by the research ethics board at the Centre for Addiction and Mental Health (REB #011-2022 and 123-2020).

### Statistical analyses

Demographic information of participants was summarised using frequencies and percentages. WEMWBS total score means and standard deviations were calculated for each variable group (e.g. average WEMWBS total score in women). Student's *t*-tests and one-way analyses of variance (ANOVAs) were conducted to identify statistically significant differences in WEMWBS total scores between categorical variable groups. Variables with statistically significant differences in WEMWBS total scores were included in the hierarchical regression model. Self-efficacy scores were converted to *z*-scores for standardisation. Preliminary analyses were conducted to determine if the selected variables met assumptions for regression analysis.

A three-step hierarchical regression was conducted to analyse the ability of survey variables to predict variability in mental well-being. The first, second and third steps added demographic, situational and psychological variables, respectively, on a stepwise basis to allow for identifying each category's contribution to the predictive power of the regression model. Through the use of a hierarchical regression model, the relative contribution of each predictor variable was quantified while simultaneously allowing for statistical control of potential confounding variables. All statistical analyses were conducted with SPSS Version 27 (IBM Corporation 2020).

## Results

### Participant demographics

Some 718 people expressed interest in the study, 294 people provided written consent to participate and 232 people filled out initial baseline survey data. Of these 232 individuals, 202 provided sufficient survey data to be included in the final study sample. Respondents that were missing more than 30% of their data were deemed ineligible from data analysis to mitigate non-response bias.^26^ Three participants were ineligible because of family member age and 27 participants were excluded because of missing response items. Excluded participants were similar demographically to those included in the study. Most of their family members were over the age of 30 and almost all lived with their family. While most of the excluded participants provided demographic information, many were missing information regarding mental well-being and the independent variables investigated in the study.

Of the included participants, 142 (70.30%) lived in Ontario and 164 (81.19%) identified as White. Eighty-eight (43.56%) were over the age of 60 and 159 (78.71%) were mothers of an adult with an IDD. Of all the adults cared for by respondents, 75 (37.13%) were over the age of 30 and 144 (71.29%) lived with their family. The mean WEMWBS total score of participants was 43.6 (s.d. = 9.9). Seventy-four caregivers (36.6%) had scores below 41, indicating risk for depression.

Of the demographic variables, WEMWBS total scores were significantly lower among women, respondents under the age of 60 and respondents who completed the survey before COVID-19 vaccines were available to the general public. In terms of situational variables, WEMWBS total scores were significantly lower in respondents who were affected ‘a lot’ by lack of programming for the adult they care for, and in respondents who defined their level of social connection with other families as ‘isolated’. Lastly, WEMWBS total scores were significantly lower in participants who rated themselves <50 in any of the four self-efficacy domains: confidence in ability to communicate effectively and prepare for healthcare for my family member, confidence in ability to support and manage the mental health of my family member, confidence in ability to appropriately manage burnout and build resilience in myself and confidence in ability to work effectively across health and social systems. Detailed comparisons of mental well-being scores based on demographic, situational and psychological variables can be found in Table 1.

**Table 1:** Differences in Warwick-Edinburgh Mental Wellbeing Scale (WEMWBS) total scores based on demographic, situational and self-efficacy variables

Variable	*n* (%)	WEBWMS mean (s.d.)
Gender		
Women	186 (92)	43.11 (9.80)*
Men	16 (8)	48.94 (9.23)*
Age		
<60	114 (56)	42.29 (9.84)*
60+	88 (44)	45.24 (9.70)*
Age of loved one		
<30	127 (63)	42.66 (9.93)
30+	75 (37)	45.12 (9.61)
Relation		
Mother	159 (79)	42.88 (9.79)
Father	13 (6)	49.69 (10.11)
Sibling	23 (11)	43.13 (9.01)
Other	7 (4)	45.14 (11.78)
Region		
Atlantic^a^	9 (5)	46.33 (9.87)
British Columbia	18 (9)	45.22 (9.51)
Ontario	142 (70)	43.25 (9.74)
Prairies^b^	27 (13)	42.63 (10.42)
Quebec	6 (3)	46.33 (12.86)
Ethnicity		
White	164 (81)	43.20 (9.84)
Other	38 (19)	45.18 (9.93)
Living situation		
With family	144 (71)	43.99 (9.46)
Independently	16 (8)	43.88 (9.63)
Group	33 (16)	42.76 (11.44)
Other	9 (5)	39.44 (11.04)
Vaccine availability		
Pre	116 (57)	41.89 (10.56)**
Post	86 (43)	45.85 (8.36)**
Lack of programming		
No/little impact	79 (39)	46.19 (9.61)**
Large impact	123 (61)	41.68 (9.69)**
Difficulty accessing healthcare		
No/little impact	144 (71)	44.42 (9.77)
Large impact	58 (29)	41.47 (9.85)
Connection to Families		
Regular/little connection	140 (69)	44.64 (9.92)*
Socially isolated	62 (31)	41.18 (9.38)*
Confidence in ability to communicate effectively and prepare for healthcare for my family member		
<50	27 (13)	34.63 (6.86)***
50+	175 (87)	44.95 (9.54)***
Confidence in ability to support and manage the mental health of my family member		
<50	73 (36)	37.73 (9.27)***
50+	129 (64)	46.88 (8.59)***
Confidence in ability to appropriately manage burnout and build resilience in myself		
<50	69 (34)	35.64 (8.19)***
50+	133 (66)	47.69 (7.97)***
Confidence in ability to work effectively across health and social systems		
<50	57 (28)	36.19 (9.11)***
50+	145 (72)	46.48 (8.57)***

a. New Brunswick, Nova Scotia, Newfoundland, Prince Edward Island.

b. Manitoba, Saskatchewan, Alberta.

*P < 0.05, **P < 0.01, ***P < 0.001.

### Regression analysis

All variables that were found to have statistically significant differences in WEMWBS total scores were included in a regression model. A three-step hierarchical regression was conducted, with the first step including sociodemographic variables, the second step adding situational variables and the third step adding psychological variables.

Age and gender (i.e. sociodemographic variables) were included as variables in the first step of the regression model. The results indicated that the model was a significant predictor of mental well-being, *F*(2,199) = 5.628, *P* = 0.004. Gender (*B* = 6.50, *P* = 0.011) and age (*B* = −3.34, *P* = 0.016) contributed significantly to the model. Overall, the model was a relatively weak predictor of mental well-being (*R* = 0.231), and predicted 5.4% of variance in mental well-being.

The second step included the aforementioned sociodemographic variables, as well as situational variables (vaccination availability, connection to other families and lack of programming). Results indicated that the model was a moderately weak predictor of mental well-being (*R* = 0.354), predicting 12.5% of the variance in well-being. Nevertheless, the model was significant, *F*(5,196) = 5.60, *P* < 0.001, with gender (*B* = 5.00, *P* = 0.047) and vaccination availability (*B* = 3.47, *P* = 0.011) contributing significantly to the model. In addition, the change in *R*^2^ from the first model to the second model was significant (Δ*R*^2^ = 0.071, *P* = 0.001), suggesting that the additional situational variables significantly improved the regression model's predictive ability.

Psychological self-efficacy scores were added in the third step. Connection to other families (*B* = −3.16, *P* = 0.003), confidence in ability to appropriately manage burnout and build resilience (*B* = 5.43, *P* < 0.001) and confidence in ability to work effectively across health and social systems (*B* = 2.36, *P* = 0.003) contributed significantly to the model. Overall, the model was significant, *F*(9,192) = 26.75, *P* < 0.001. It was a moderate predictor of mental well-being (*R* = 0.746), and it predicted 55.6% of variance in mental well-being. In addition, the change in *R*^2^ from the second to the third model was significant (Δ*R*^2^ = 0.431, *P* < 0.001), suggesting that the additional variables significantly improved the regression model's predictive ability.

The full list of unstandardised coefficients for variables predicting mental well-being based on the WEMWBS score can be found in Table 2.

**Table 2:** Hierarchical regression model analysis

Variable	*B*	s.e.	*P*	*B*
				95% CI [LL, UL]
Model 1				
(Intercept)	44.94	1.03	<0.001***	[42.90, 46.98]
Gender	6.50	2.53	0.011*	[1.51, 11.48]
Age (<60 *v.* 60+)	−3.34	1.38	0.016*	[−6.05, −0.62]
Model 2				
(Intercept)	45.59	1.47	<0.001***	[42.69, 48.50]
Gender	5.00	2.50	0.047*	[0.74, 9.92]
Age (<60 *v.* 60+)	−2.46	1.35	0.071	[−5.12, 0.21]
Vaccination availability	3.47	1.35	0.011*	[0.81, 6.13]
Connection with other families	−2.82	1.46	0.055	[−5.69, 0.06]
Lack of programming	−2.70	1.41	0.056	[−5.47, 0.07]
Model 3				
(Intercept)	45.57	1.06	<0.001***	[43.48, 47.66]
Gender	2.44	1.83	0.184	[−1.17, 6.06]
Age (<60 *v.* 60+)	−1.20	0.98	0.223	[−3.15, 0.74]
Vaccination availability	−1.23	1.04	0.236	[−3.28, 0.82]
Connection with other families	−3.16	1.05	0.003**	[−5.23, −1.09]
Lack of programming	−0.03	1.04	0.977	[−2.09, 2.02]
Self Efficacy-1: Confidence in ability to communicate effectively and prepare for healthcare for my family member	0.77	0.77	0.324	[−0.76, 1.19]
Self Efficacy-2: Confidence in ability to support and manage the mental health of my family member	−0.79	0.84	0.351	[−2.45, 0.87]
Self Efficacy-3: Confidence in ability to appropriately manage burnout and build resilience in myself	5.43	0.69	<0.001***	[4.07, 6.78]
Self Efficacy-4: Confidence in ability to work effectively across health and social systems	2.36	0.77	0.003*	[0.83, 3.88]

CI, confidence interval; LL, lower limit; UP, upper limit.

*P < 0.05, **P < 0.01, ***P < 0.001.

## Discussion

This study investigated the association of demographic, situational and psychological variables with mental well-being among family caregivers of adults with IDDs that were taking part in a virtual course to support mental health and well-being. Participants were recruited from provincial and national developmental disability organisations across Canada.

To assess which variables contributed to mental well-being, as measured by participants’ WEMWBS total score, hierarchical regression analysis was used wherein the first model included demographic variables; the second model included demographic and situational variables; and the third model included demographic, situational and psychological variables. Results indicated that the first model predicted 5.4% of variance in well-being scores, the second model predicted 12.5% of variance and the third model predicted 55.6% of variance. In the final model, the only variables that contributed significantly to predicting variance in mental well-being scores among family caregivers were psychological in nature: connection to other families, confidence to manage burnout in oneself and confidence in ability to work effectively across social systems. This suggests that when addressing mental health concerns in this population, particularly during public health emergencies, there may be value in taking an approach that focuses on service navigation and addressing burnout, in addition to exploring opportunities to help families feel more connected with one another.

The results of the present study suggested that psychological self-efficacy is an important contributor to mental well-being. This is in keeping with existing literature.^27,28^ In this study, caregivers rated their capacity in four areas, two of which significantly contributed to predicting mental well-being: confidence to manage burnout in oneself and confidence in ability to work effectively across health and social care systems. Research done before the pandemic has highlighted the challenges that families face related to service navigation and the need to improve the process.^29,30^ These challenges have been exacerbated since the start of the pandemic and have taken a major toll on families because of service disruption, the switch of services to online platforms and uncertainty of where to go.^4,16,18,19^ However, previous studies that teach families how to navigate these systems were effective,^31^ suggesting that programmes that empower family caregivers to navigate health and social systems are valuable for this population.

The other self-efficacy variable that significantly contributed to predicting mental well-being was confidence to manage burnout in oneself. Whether it is called burnout or compassion fatigue, many families report exhaustion, especially as caring demands increase over time.^13,16,32^ Participants on the course acknowledged that they were not paying as much attention to themselves as they could be, and that it was a challenge to do so when demands both related and unrelated to caregiving were high.^22^ Therefore, it is important for providers to assess caregivers’ well-being to help identify their mental health needs, and to put measures in place if they feel overwhelmed or are in distress. The importance of ensuring family caregivers of people with IDDs feel supported and of mitigating caregiver distress has been emphasised in National Institute for Health and Care Excellence (NICE) guidelines, as well as the Canadian guidelines on developmental disabilities for primary care providers.^1,33,34^ This is likely even more crucial in pandemic recovery, given the trauma individuals and their families have endured, the persistence of service disruptions and the reality that the health and situations of some people with IDDs have not improved despite the resolution of the COVID-19 pandemic.^35^

Another significant variable was participants’ sense of connection with other families. This was the only situational variable that contributed significantly to predicting mental well-being in the third model that included all variables. Interestingly, this variable was not significant in the second step but was significant in the third. It is likely that the addition of self-efficacy scores significantly improved the predictive strength of this variable by suppressing errors.^36^ There is substantial evidence that formal and informal peer connections have a positive impact on well-being,^37,38^ and provide opportunities for caregivers to feel less alone in their circumstances.^39^ However, pandemic-associated programme disruptions resulted in a loss of valuable informal connections.^2,16^ Given the results of this study and existing literature, social connection is likely an important intervention area for this group. It would be important to explore the benefits of social connection not only during emergencies, but also as families move forward during pandemic recovery and navigate healthcare systems still affected by unresolved problems that emerged because of the pandemic.

While changes in public health rules and availability of measures such as vaccines can significantly affect the mental health of the general population,^40^ it is important to note that variables such as vaccination availability did not significantly contribute to predicting well-being when combined with other variables in this study. Demographic variables, such as gender and age, also did not significantly predict mental well-being when combined with other variables. This suggests that mental well-being goes far beyond family role, age or gender.

There are several limitations to this study. First, it is not possible to establish causation between variables using multiple regression and a cross-sectional study design. Therefore, it is impossible to know if significant contributors to prediction, such as connection to other families, directly caused changes to mental well-being. Second, the study population was a convenience sample. It consisted largely of people with access to technology, and enough resources and time to participate in an intervention-based study during the pandemic. This study did not include people who did not feel that such a course was needed, nor people unable to engage in this type of learning because of caregiving responsibilities or other factors. Moreover, study participants were recruited from 16 national and provincial developmental disability groups, as well as partner agencies. Through recruiting from a broad variety of organisations, we hoped to capture a diverse study sample that was representative of the general population. However, participants were predominantly Caucasian women with the capacity to participate in this online intervention. More work is needed to investigate the mental well-being of caregivers specifically from underrepresented groups. Objective grading of the severity of IDD diagnosis of family members that participants cared for was not obtained, and thus results could not be stratified by diagnosis severity. Lastly, at the time of writing there were no Canadian studies that used the WEMWBS scale to describe the mental well-being of caregivers of adults with IDDs before the pandemic. Therefore, it is not possible to draw a direct comparison in mental well-being before and during the COVID-19 pandemic using the WEMWBS scale.

Pre-pandemic normalcy had not returned for the families of adults with IDDs when they completed these surveys between fall of 2020 and summer of 2022. There was a demonstrated need to balance attention on reducing the risk of infection with promoting mental well-being in this group. Researchers and care providers for this population may need to tailor management approaches to match family needs as they evolve over time, including during and after public health emergencies. The results from this study suggest that service navigation supports, fostering social connections and managing burnout could have been valuable intervention targets for improving mental well-being in family caregivers of adults with IDDs during the pandemic and perhaps also during pandemic recovery.

## Data Availability

The anonymised data that support the findings of this study are available upon reasonable request from the corresponding author, Y.L.
